# Case report: Epithelioid inflammatory myofibroblastic sarcoma treated with an ALK TKI ensartinib

**DOI:** 10.3389/fonc.2023.1084456

**Published:** 2023-03-22

**Authors:** Mengmeng Li, Ruyue Xing, Jiuyan Huang, Chao Shi, Chunhua Wei, Huijuan Wang

**Affiliations:** ^1^ Department of Medical Oncology, The Affiliated Cancer Hospital of Zhengzhou University and Henan Caner Hospital, Zhengzhou, China; ^2^ Department of Molecular Pathology, The Affiliated Cancer Hospital of Zhengzhou University and Henan Cancer Hospital, Zhengzhou, China

**Keywords:** epithelioid inflammatory myofibroblastic sarcoma, inflammatory myofibroblastic tumor, *RANBP2-ALK*, ensartinib, fluorescence *in situ* hybridization

## Abstract

Epithelioid inflammatory myofibroblastic sarcoma (EIMS) is an aggressive variant of inflammatory myofibroblastic tumor (IMT) and has a poor prognosis. EIMS is characterized by epithelioid morphology, neutrophilic infiltrate and specific fusion partners of anaplastic lymphoma kinase (*ALK*). Despite no standard therapy for EIMS, ALK tyrosine kinase inhibitors (TKIs) are recommended for these tumors. The present case describes an abdominal mass that presented in a 31-year-old male. The patient suffered from recurrence and multiple metastases 2 months after surgery. Ensartinib was administered and *RANBP2-ALK* fusion was detected. A partial response has been observed for 4 months and there has been no recurrence. This study provided a successful case with sustained response of targeted therapy.

## Introduction

Inflammatory myofibroblastic tumor (IMT) is as an intermediate soft tissue tumor composed of myofibroblast-differentiated spindle cells along with numerous inflammatory cells, plasma cells, and/or lymphocytes (1). Epithelioid inflammatory myofibroblastic sarcoma (EIMS) is a rare subtype of IMT that is characterized by aggressiveness, rapid local recurrence, earliest metastasis, and fatality (2, 3). EIMS differs from the conventional spindle-cell IMT in that it consists mostly of round-to-epithelioid cells, with a loose or myxoid stroma that is infiltrated with abundant neutrophils (4–7). Among 11 cases, all tumors were located in the abdomen and most originated in the mesentery or omentum (2). In addition to abdomen, several cases with extra-abdominal sites of EIMS have been reported, including liver (8), lung (7, 9), pericardium (10), ovary (11), cutaneous (12), stomach (13), groin (14) and central nervous system (15). A variety of gene partners have been observed in IMT including NPM, TMP3/4, CARS, CLTC, EML4, DCTN1, SEC31L1, ATIC and FN1 (5), with more prevalent fusions of RANBP2 (2), RRBP1 (16) and EML4 (5) in EIMS. Furthermore, G1269A has been reported as a secondary mutation after resistance to crizotinib (17). As described herein, a patient with *RANBP2-ALK* EIMS in the greater omentum benefited from an ALK TKI.

## Case description

**Figure 1:**
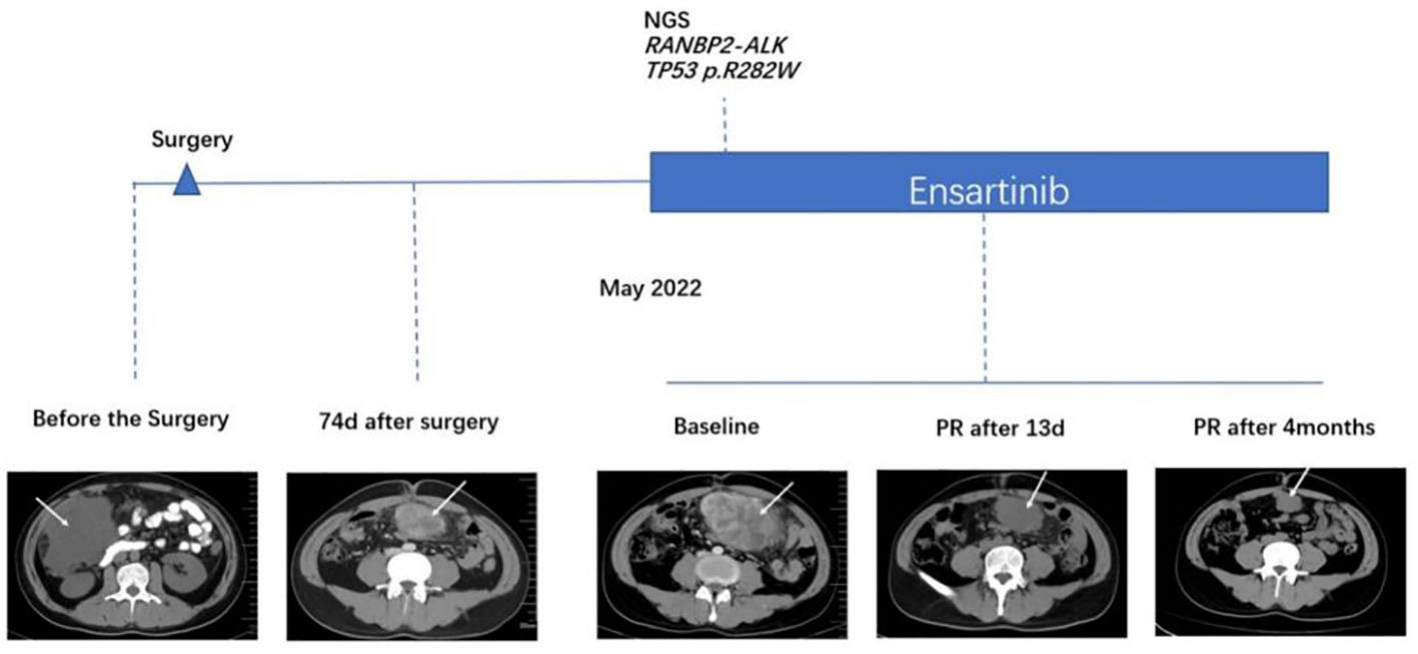
Comparison of computed tomography images before and after treatment with ensartinib.

**Figure 2:**
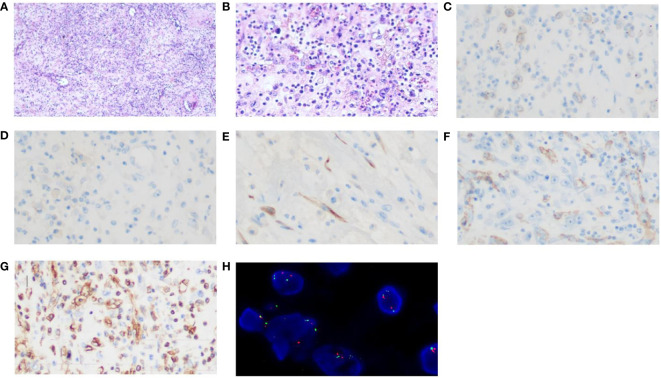
Histopathological examination with haematoxylin and eosin staining and Immunohistochemistry and FISH images. The lesion consisted of both epithelioid and spindle cells with enlarged nucleolus and inflammatory cells infiltration **(A)** (magnification, x40). **(B)** (magnification, x200). **(C)** ALK positivity (magnification, x400); **(D)**Focal CD30 weakly positive (magnification, x400); **(E)**Desmin positivity (magnification, x400) **(F)**SMA positivity (magnification, x400) **(G)** Vimentin positivity (magnification, x400); **(H)** A FISH image of ALK rearrangement.

An intermittent abdominal pain and abdominal distention were reported by a 31-year-old Chinese male. Enhanced computed tomography (CT) scans of the abdomen revealed an abdominal mass that was suspected to be gastric stromal tumor (**Figure 1** and **Table 1**). Biopsy showed a loose tissue composed of spindle cells, hollow cells and small blood vessels. Fluorescence *in situ* hybridization (FISH) was negative for CHOP and MDM2, excluding the presence of liposarcoma. Abdominal tumor resection plus partial colectomy was then performed in March 2022, removing the greater omentum tumor as well as partial transverse colon and ascending colon with a volume of 12 × 10 × 10 cm. The histopathological results revealed the lesion contained both epithelioid and spindle cells with enlarged nuclei and infiltrating inflammatory cells, primarily plasma cells, eosinophils, neutrophils, lymphocytes (**Figures 2A, B**). EIMS usually expressed vimentin and desmin positively, the Expression situation of EMA, CD30 and SMA was inconsistent. EIMS can be distinguished by IHC from soft tissue tumors with epithelioid cell morphology and tumors with significant mucoid background. Such as anaplastic large cell lymphoma (ALCL) and EIMS were positive for SMA, CD30 and ALK and negative for EMA (29). But no desmin was found in ALCL. Follicular dendritic cell sarcoma (FDCS): Immunohistochemical expression of CD21, CD23, or CD35 was positive, but no ALK,Desmin,WT-l,orD2-40 (30). Extragastrointestinal stromal tumor (EGIST) CD117, CD34 and Dog-1 were positive, and ALK;CKDesmin were negative (31). Therefore, the following markers were selected for immunohistochemistry, and the results were as follows. ALK, Vimentin, Desmin, SAM, Ki-67, CD30, CD31, Catenin and H3K27Me3 were positive, while negative for Cytokeratin (CK), CD34, CD117, S-100, SOX-10, Dog-1, Muc-4, EMA and ERG (**Table 2**). Antibody clone of ALK-IHC is ALK p80(5A4), and Positive immunohistochemical results of ALK, CD30, desmin, SAM and Vimentin are shown in turn in this **Figures 2C–G**. Approximately 18% of the tumor cells showed a rearrangement of ALK by FISH (**Figure 2H**). These findings are in line with those of EIMS. Adjuvant therapy was not administered to the patient following surgery.

**Table 1:** Clinicopathological characteristics of EIMS in reported cases and our case.

Case/Reference	Age/ Sex	Site	Symptom	Size (cm)	Multifocal	Treatment	Specific drug names of ALK-TKIs	Response at TKIs	PFS at TKIs (m)	Follow-up (m)
1 (18)	44/M	Omentum	early satiety and abdominal pain	NA	Y	SE+CT+ALKi→ALKi→ALKi	Imatinib→Crizotinib→Crizotinib	NA→PR→PR	3→8→19 (AWD)	40 (AWD)
2 (2)	41/M	Omentum	NA	26	Y	SE+CT+ALKi	NA	NA	NA	40 (ANED)
3 (19)	57/M	Pleura or chest wall	dyspnea on exertion	NA	NA	ALKi	NA	NA	NA	NA
4 (20)	22/M	the ileum	fever, epigastralgia	5.5×6	Y	SE+CT+ALKi	crizotinib	PR	10(AWD)	17 (AWD)
5 (7)	21/M	the left lower lobe of lung	general fatigue and rapid weight loss	10	Y	SE+ALKi	crizotinib	NA	4	4(STD)
6 (21)	71/M	the lung and pleural-based mass	dyspnea on exertion and weight loss	12.5×12×8	Y	SE+CT+ALKi →ALKi	crizotinib→NA	NA→PR	2→9 (AWD)	12(AWD)
7 (22)	16/F	the lung	NA	8	NA	SE+CT+RT+ALKi	crizotinib	PR	19 (AWD)	33 (AWD)
8 (23)	22/M	the transverse colon mesentery	abdominal pain and fever	13	NA	SE+ALKi	crizotinib	NA	16 (AWD)	17(AWD)
9 (24)	22/M	Mesentery of colon	abdominal pain and fever	20×15	Y	SE+ALKi	crizotinib	PR	12 (AWD)	14(AWD)
10 (5)	45/M	omentum	abdominal distention and abdominal pain	20	Y	SE+ALKi	crizotinib	NA	0.5	2(STD)
11 (11)	15/F	Ovary	NA	NA	Y	CT+SE+ALKi	crizotinib/ceritinib	PR	NA	24(AWD)
12 (17)	26/M	Abdomen	fever, abdominal distention	NA	Y	ALKi→ALKi	crizotinib→brigatinib	PR→PR	9→8(AWD)	24(AWD)
13 (25)	46/F	Abdomen	abdominal pain,abdominal distention	11×6.5×7	NA	SE+ALKi+CT	crizotinib	PR	2	14(STD)
14 (26)	NA	colon sigmoideum	NA	11.9×6.9	NA	SE+ALKi	crizotinib	NA	NA	NA
15 (15)	72/F	brain	NA	4.7×1.6×1.2	N	SE+ALKi	Alectinib	NA	NA	NA
16 (27)	42/F	omentum	abdominal distention and abdominal pain	19×19×10	Y	SE+ALKi→ALKi→ALKi→ALKi	Crizotinib→Alectinib→Ceritinib→Lorlatinib	PR→PR→PR→SD	5→5.5→6→>5(AWD)	>24(AWD)
17 (28)	14.7/M	pelvis	Ascites, pleural effusions	NA	N	ALKi	crizotinib→crizotinib	CR→PR	5→17	NA
18 (28)	11.3/M	Abdomen mesenteric	NA	NA	Y	SE+ALKi	crizotinib	PR	48 (ANED)	48 (ANED)
19 (28)	9.1/M	Abdomen mesenteric	Massive ascites, abdominal compartment syndrome	NA	Y	ALKi→ALKi	crizotinib→ceritinib+CT	CR→PR	11→6	23(STD)
20 (28)	1.4/F	Abdomen mesenteric	NA	NA	Y	ALKi	crizotinib	CR	11(AWD)	11(AWD)
**Current case**	**31/M**	**Abdomen**	**abdominal distention and abdominal pain**	**12×10×10**	**Y**	**SE+ALKi**	**Ensartinib**	**PR**	**4(AWD)**	**4(AWD)**

F, female; M, male; Y, yes; N, no; m, month; cm, centimeter; PFS, progression-Free-Survival; ALKi, ALK inhibitor; ANED, alive, no evidence of disease; AWD, alive with disease; STD, succumbed to disease; CT, chemotherapy; NA, data not available; SE, surgical excision; CR, complete response; PR, partial respond; SD, stable disease.

The bold values indicate the current case.

**Table 2:** Immunohistochemical and genetic characteristics in reported cases and our case.

Case	Vimentin	DES	SMA	CD30	CK	EMA	MYF4	DOG 1	S-100	ALK			
										IHC	FISH	RT-PCR	NGS
1	NA	+	–	NA	–	NA	–	NA	NA	NM	ALK-rearrangement	RANBP2	NA
2	NA	+	–	+	–	–	–	NA	–	NM	ALK-rearrangement	RANBP2	NA
3	+	+	–	–	–	NA	–	NA	–	PN	RANBP2	NA	NA
4	NA	+	+	NA	NA	NA	–	NA	–	NM	NA	RANBP2	NA
5	+	+	–	–	–	NA	–	NA	–	CN	ALK-rearrangement	NA	NA
6	NA	NA	NA	NA	NA	NA	NA	NA	NA	NA	ALK-rearrangement	NA	NA
7	NA	NA	NA	+	NA	NA	NA	NA	NA	CN	ALK-rearrangement	NA	NA
8	NA	+	–	+	–	NA	NA	–	–	PN	RANBP2	NA	NA
9	NA	+	–	–	–	–	–	NA	–	CN	ALK-rearrangement	NA	NA
10	NA	+	+	NA	NA	NA	NA	NA	NA	CN	NA	EML4	NA
11	NA	+	+	NA	NA	NA	NA	NA	NA	NM	NA	NA	RANBP2
12	NA	NA	–	NA	–	–	–	NA	NA	NA	ALK-rearrangement	NA	RANBP2
13	+	NA	+	NA	NA	NA	NA	–	–	NA	NA	NA	NA
14	NA	+	–	+	–	–	NA	–	–	NA	RRBP2	NA	NA
15	NA	+	–	–	–	+	NA	NA	–	CN	NA	VCL	NA
16	NA	+	–	NA	–	–	NA	NA	–	NA	ALK-rearrangement	NA	PRRC2B
17	NA	NA	NA	+	NA	NA	NA	NA	NA	NM	ALK-rearrangement	RANBP2	NA
18	NA	NA	NA	+	NA	NA	NA	NA	NA	NM	ALK-rearrangement	RANBP2	NA
19	NA	NA	NA	+	NA	NA	NA	NA	NA	NM	ALK-rearrangement	RANBP2	NA
20	NA	NA	NA	+	NA	NA	NA	NA	NA	NM	ALK-rearrangement	NA	NA
**Current case**	**+**	**+**	**+**	**+**	**-**	**-**	**NA**	**-**	**-**	**NA**	**ALK-rearrangement**	**NA**	**RANBP2**
Total	100%	100%	33%	69%	0%	13%	0%	0%	0%				
	(4/4)	(13/13)	(5/15)	(9/13)	(0/9)	(1/8)	(0/7)	(0/4)	(0/11)				

+, positive cells; −, negative staining; NM, nuclear membrane staining; PN, cytoplasmic staining with perinuclear accentuation; CN, cytoplasmic pattern; CK, cytokeratins; DES, desmin; EMA, epithelial membrane antigen; FISH, fluorescence in situ hybridization; MYF4, myogenin; CD, cluster of differentiation; DOG-1, anoctamin−1; NGS, next-generation Sequencing; NA, data not available; RT−PCR, reverse transcription−polymerase chain reaction; SMA, smooth muscle actin; IHC, immunohistochemistry; ALK, anaplastic lymphoma.

The bold values indicate the current case.

The patient presented to our hospital with abdominal distension 74 days after surgery. CT showed new soft tissue mass with peritoneal metastasis in lower abdomen and pelvis, indicating tumor recurrence. The tumor was significantly enlarged 14 days later by CT scan. The therapeutic course and radiological examinations of the patient were summarized in **Figure 1**. The patient was subsequently treated with ensartinib (225 mg, QD). The CT revealed notable tumor shrinkage 13 days after ensartinib treatment. In September 29, 2022, 4 months after initiation of ensartinib, this patient still achieved partial response (PR) according to the Response Evaluation Criteria in Solid Tumors (RECIST) version 1.1 (**Figure 1**). Next-generation sequencing (NGS) was employed using a 1021-gene panel (Burning Rock, Guangzhou, China). NGS identified *RANBP2-ALK* fusion (EX18:EX20) with mutational abundance of 2.3% and a TP53 p.R282W mutation with a mutational abundance of 3.2% using the biopsy specimen of the abdominal mass (data not shown). As we prepared the manuscript, the patient remained PR and continued to receive ensartinib without any significant adverse events.

## Discussion

EIMS is first named in 2011 (2) and is a more aggressive subtype of IMT and characterized by epithelioid-to-round cell morphology and prominent inflammatory infiltrate. EIMSs can occur across a wide age range (4 months to 76 years), with a male and intra-abdominal predominance (15). Recently, IMTs associated with *EML4-ALK* have been classified as EIMS (5).

EIMS is essentially a histopathological diagnosis. Even with auxiliary detection, it is difficult to make a definitive diagnosis of EIMS on a small biopsy due to its genetic overlap with other ALK-positive tumors. To date, more than 40 cases of EIMS have been reported, among which 20 cases (2, 5, 7, 11, 15, 17–28) as well as present case with the complete clinicopathological, immunohistochemical and genetic characteristics were summarized in **Table 1**, **2**. These 21 cases were composed of 14 (67%) adults (21-72 years old) and 6 (29%) children or adolescents. The median age was 24 years old. Fourteen (67%) patients were male while six (29%) were female. In 16 patients, the tumors were found in abdominal cavity (omentum, mesenterium, ileum, colon, ovary, etc.), 4 in pleural cavity and 1 in brain, which consisted with previous reports that EIMS had a male and intra-abdominal predilection. In contrast, conventional spindle-cell IMT was slightly more prevalent among females. The EIMS exhibits distinctive morphological characteristics including loosely arranged round or epithelioid neoplastic cells with vesicular nuclei, prominent nucleoli and myxoid stroma surround by amphophilic to eosinophilic cytoplasm. Neutrophil-rich inflammatory infiltrates are a striking characteristic of EIMS. Almost all tumors contained a spot of spindle cell component.

According to immunohistochemical results in 15 cases with EIMS, 8 (53%) revealed a unique nuclear membrane staining pattern for ALK (**Table 2**). Nevertheless, cytoplasmic or perinuclear staining of ALK was observed in 7 of 15 cases (47%). All cases (13/13) exhibited strong expression of desmin, another diagnostic immunophenotype. Besides, the tumor displayed variable expression of CD30 (69%, 9/13), alpha smooth muscle actin (33%, 5/15) and epithelial membrane antigen (13%, 1/8). Moreover, all cases were negative for cytokeratin (0/9), myogenin (0/7), anoctamin−1 (0/4) and S-100 (0/11). Of note, FISH assay, PCR assay or NGS can contribute to the diagnosis of EIMS. It has been confirmed that ALK rearrangement is present in 16 cases by FISH, 8 cases by PCR and 4 cases by NGS.

*RANBP2-ALK* and *RRBP1-ALK* fusions were the most reported driver mutation of EIMS (16, 32). In previous studies as well as our case with EIMS, nuclear membrane or perinuclear staining pattern for *RANBP2-ALK* fusion was detected, and almost all cases containing *RANBP2-ALK* fusion exhibited aggressive behavior (2, 12, 18, 20, 33–35). Despite of the unclear biological function, the chimeric *RANPB2-ALK* gene was reported to promote cell growth and proliferation regardless of cytokine *in vitro (* 36, 37). There was a specificity for *RRBP1-ALK* in EMIS with cytoplasmic ALK expression and clinically aggressive progression, suggesting that *RRBP1-ALK* may exert relapsed oncogenic role in clinically aggressive EIMS (16). Recently, *PRRC2B-ALK* fusion was also considered as the main oncogenic driver of the EIMS (27).

There is no clear consensus on the best treatment for EIMS. The main treatment option remains surgical resection. Postoperative adjuvant therapy has not yet been identified due to the limited available experiences. In terms of rapid recurrence, postoperative chemotherapy or radiotherapy appeared to exert finite effect (2, 18, 20, 35). Crizotinib has been applied to treat EIMS in several cases and showed favorable efficacy (2, 18–20, 38). In our summarized cases, 10 patients continued to live with disease with follow-up of 11-40 months. Among 21 patients, 17 received crizotinib, apart from 2 unknown TKIs, 1 ensartinib and 1 alectinib. Fifteen of 17 patients who received crizotinib had survival information, with a median PFS of 9 months and mean PFS of 10.8 months. Furthermore, a case with *PRRC2B-ALK* fusion showed durable clinical response to sequential use of ALK TKIs (crizotinib, alectinib, ceritinib and lorlatinib) (27). The development of drug resistance to ALK TKIs is a major issue. A previous case revealed that R1192P was found as a resistance mutation to crizotinib (27), suggesting that the application of NGS was important to identify actionable mutations and resistance mechanisms. This may contribute to molecular targeted therapies for EIMS with *ALK* gene arrangement. Considering the broader coverage of targets and stronger tissue penetration, we speculated that patients may have more benefits from the direct second- or third-generation ALK TKIs compared with the sequential treatment of ALK TKIs. Therefore, in our own case, the patient received ensartinib (a second-generation ALK TKI) post relapse. After four months of treatment, the patient felt well with PR, and follow-up CT scan showed that the residual tumor was partially shrank.

Recently, a diffuse positive signal was observed in EIMS for programmed death-ligand 1 (PD-L1) (4), providing a possible inmmunomodulatory therapies targeting the PD-1/PD-L1 pathway. Moreover, CD30 appeared to commonly express in EIMS in our summarized cases (**Table 1**). The survival time was prolonged by ALK and CD30 combination therapies (39). With increasing evidence of EIMS, molecular mechanisms will be clear and potential treatments will be developed in the future.

In addition to *RANBP1-ALK* fusion, TP53 p.R282W mutation was also identified in our case. Mutant TP53 is closely related to the occurrence, development and prognosis of tumor (40, 41). Besides, mutations in TP53 independently promoted metastasis, decreased TKI responses and shorten overall survival in ALK-positive lung adenocarcinoma (42). However, the molecular pathological importance of the TP53 mutation in IMT has not been elucidated thus far. A previous study demonstrated that abnormal TP53 staining patterns were detected in only approximately 7% of IMT, with TP53 missense mutations occurring in 13% of cases, suggesting that TP53 mutation in IMT was an infrequent event and may not attribute to its pathogenesis (43). Recently, a study demonstrated that ORR of ensartinib was high regardless of TP53 mutation status (44). In our case, ensartinib also showed favorable efficacy.

## Conclusion

In conclusion, we described a typical EIMS case with a round or epithelioid morphology of cells, accompanied by a high relapse and a poor prognosis. To the best of our knowledge, our report is the first case to investigate the efficacy of ensartinib for EIMS. The clinical management and results of the patients were introduced in detail in our case; besides, the pathological and genetic characteristics of the tumors were reviewed. By analyzing the efficacy of ALK TKIs in EIMS in previous literature, we found that ALK TKIs are effective for EIMS treatment. However, given the lack of the clear resistance mechanisms, further research is needed. Detection of *ALK* rearrangement is essential for correct diagnosis of EIMS and provides a fundamental basis for ALK TKI therapy.

## Data availability statement

The original contributions presented in the study are included in the article/supplementary material. Further inquiries can be directed to the corresponding author.

## Ethics statement

The studies involving human participants were reviewed and approved by the Ethics Committee of Henan Cancer Hospital. The patients/participants provided their written informed consent to participate in this study. Written informed consent was obtained from the individual(s) for the publication of any potentially identifiable images or data included in this article.

## Author contributions

>HW contributed to the conception of the study. ML and RX integrated all information and wrote the main manuscript. CW supervised the writing process. CS and JH provided critical guidance. All authors contributed to the article and approved the submitted version.
